# Comparative Characterization of Lipid Composition and Minor Components in Coffee Oils from Arabica and Robusta Spent Coffee Grounds

**DOI:** 10.3390/foods15122129

**Published:** 2026-06-12

**Authors:** Wei Zeng, Song Liao, Cheng Zhen, Meijun Du, Jun Jin, Bin Hu

**Affiliations:** 1 School of Basic Medicine, Gannan Medical University, 1 Hexie Avenue, Ganzhou 341000, China; leow.zeng@foxmail.com (W.Z.);; 2 State Key Laboratory of Food Science and Resources, School of Food Science and Technology, Jiangnan University, Wuxi 214122, China; 3 Food Laboratory of Zhongyuan, Luohe 462300, China

**Keywords:** spent coffee grounds, coffee oil, lipid composition, roasting, minor components

## Abstract

Coffee oil, an increasingly recognized yet underutilized byproduct of spent coffee grounds, has attracted attention due to its diverse lipid composition and minor components. This study systematically investigated the lipid characteristics of coffee oils extracted from both Arabica and Robusta spent coffee grounds subjected to varying roasting degrees. Comprehensive analyses were conducted, mainly regarding oil yield, acid and peroxide values, fatty acid profiles, *sn*-2 positional fatty acid distribution, triacylglycerol composition, tocopherol content and total Folin-reactive compounds, as well as squalene and sterol profiles. The selected Arabica samples generally showed higher oil yields than Robusta samples, with oil contents ranging from 12.13% to 15.14% and 10.10% to 13.01%, respectively. Arabica coffee oils showed relatively high total tocopherol levels, ranging from 930.35 to 1495.37 mg/kg, whereas Robusta coffee oils ranged from 637.69 to 867.21 mg/kg. Total Folin-reactive compounds varied among samples and should be interpreted as composition-related indicators rather than direct evidence of antioxidant function. In contrast, Robusta coffee oils contained much higher levels of squalene and total sterols, ranging from 97.00 to 170.37 mg/100 g and 787.29 to 1007.92 mg/100 g, respectively. Chemometric analyses showed distinct grouping patterns among the selected coffee oil samples. In the present sample set, the overall lipid profiles were more closely associated with the Arabica and Robusta sample groups than with the assigned roasting levels. These results provide compositional information for the potential use of Arabica coffee oil as a tocopherol- and Folin-reactive compound-rich lipid ingredient. Robusta coffee oil may be further evaluated for applications requiring higher levels of squalene, phytosterols, and relatively saturated lipid structures. This study provides novel insights into the compositional complexity of coffee oil and supports its targeted valorization across various industries.

## 1. Introduction

Coffee is the second most consumed beverage globally, after tea, and is classified as one of the three major drinks in the world, alongside cocoa and tea [1]. Its origin lies in the subtropical regions of Africa and some small islands in South Asia. Today, coffee is widely cultivated across all continents. Coffee belongs to the Rubiaceae family and the Coffea genus, with over 60 known species, though only three varieties are commercially cultivated: Arabica (*Coffea arabica*), Robusta (*Coffea canephora*), and Liberica (*Coffea liberica*) [2]. Arabica coffee is valued for its superior flavor quality and is commonly used in premium coffee products such as specialty coffee, espresso, and pour-over coffee. Robusta, on the other hand, is widely used in instant coffee and low-cost coffee beverages due to its higher caffeine content and lower production costs. Liberica coffee, with less desirable flavor and low yield, is primarily found in West Africa and is rare in the international market [3].

According to data from the United States Department of Agriculture, global coffee production for the 2025/2026 season is estimated to reach approximately 179 million 60 kg bags, with Arabica accounting for 97 million bags and Robusta for 82 million bags [4]. Spent coffee grounds (CGs) are the solid residues generated during coffee brewing after roasted and ground coffee is extracted. It is estimated that every ton of green coffee beans produces approximately 650 kg of spent CGs. Despite the rich organic content of these spent CGs, most are either incinerated, discarded, or used as low-value fertilizers, resulting in approximately 6.98 million tons of coffee waste being underutilized annually. Since brewing coffee does not extract all of its components, significant amounts of organic matter, including cellulose, hemicellulose, lignin, proteins, and lipids, remain in the CGs, with lipid content ranging from 10% to 20% [5]. This makes CGs a promising source for oil extraction.

Coffee oil, a unique and versatile natural plant oil, has garnered increasing attention in recent years due to its rich lipid composition and minor components [6]. The primary lipid form in coffee oil is triglycerides, which are composed of glycerol and three fatty acids. The type and ratio of fatty acids play a decisive role in determining the melting point, oxidative stability, and nutritional function of the oil [7]. Studies have shown that coffee oil is rich in both saturated and unsaturated fatty acids, primarily palmitic acid, stearic acid, oleic acid, and linoleic acid, making its physicochemical properties comparable to those of traditional edible vegetable oils [8,9]. In addition to fatty acids, coffee oil contains a wealth of non-saponifiable components, such as plant sterols, tocopherols, and polyphenolic compounds [8]. Plant sterols, as important components of plant cell membranes, are well-documented for their ability to lower serum cholesterol and improve cardiovascular health. Tocopherols are important antioxidant-related constituents in plant oils and are associated with the inhibition of lipid peroxidation [10]. Phenolic compounds, such as chlorogenic acid and caffeic acid, may contribute to the antioxidant-related chemical profile of coffee-derived products, although their functional activity requires direct evaluation [11,12]. Due to its diverse lipid composition and minor components, coffee oil may have potential for further development in food processing, cosmetics, and related applications.

Although the lipid characteristics and minor components of coffee oil have gained increasing attention, research on the lipid composition of coffee oil remains limited, particularly in terms of a systematic understanding of its fatty acid profile. Therefore, clarifying the lipid composition of coffee oil not only helps advance its diverse applications in food, cosmetics, and pharmaceuticals but also holds significant academic and practical value.

This study aims to provide an in-depth analysis of the lipid characteristics of Arabica and Robusta CGs from different origins and roasting degrees. Specifically, the study investigated oil content, fatty acid composition, triglyceride and sterol composition, tocopherols, total Folin-reactive compounds, and physicochemical indices such as acid value and peroxide value. This study aimed to compare the lipid composition and selected minor components of coffee oils from the selected commercial samples, providing compositional information for their further utilization.

## 2. Material and Methods

### 2.1. Reagents

Boron trifluoride, squalene, 5α-cholestan-3β-ol, gallic acid, and sterol and tocopherol standards were purchased from Sigma-Aldrich (St. Louis, MO, USA). All other reagents, including n-hexane, ethanol, diethyl ether, methanol, acetone, and acetonitrile, were obtained from Adamas Beta Shanghai Chemical & Reagents Co. (Shanghai, China). All chemicals were of analytical reagent grade, and solvents such as n-hexane, acetone, acetonitrile, methanol, ethanol, and 2-propanol were of HPLC grade.

### 2.2. Preparation of Coffee Grounds

**Figure 1:**
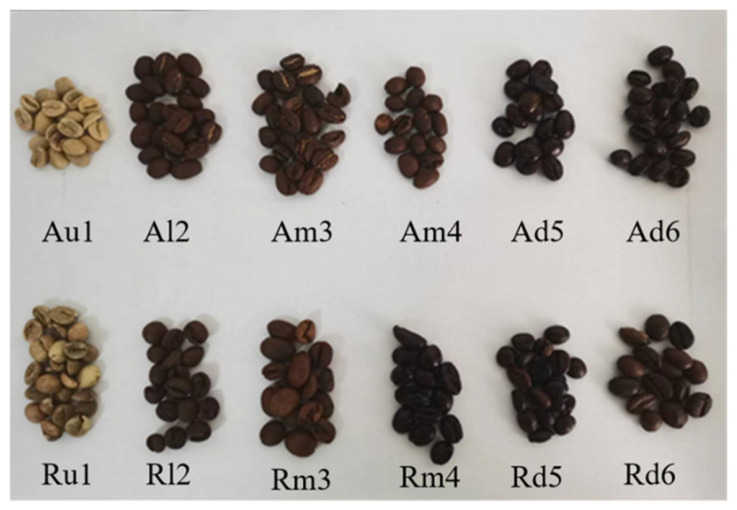
Twelve coffee bean samples.

Twelve different coffee bean samples (Figure 1) were selected, including both Arabica (*Coffea arabica*) and Robusta (*Coffea canephora*) beans with varying roasting degrees. Detailed information regarding the variety, brand, origin, and roasting degree of each sample is provided in Table 1. Because these samples were obtained from commercial sources, differences in brand, geographical origin, and commercial processing history may also contribute to the compositional variations observed among the samples. After procurement, the beans were ground and sieved using a 40-mesh screen. A mixture of 100 g of coffee powder and 150 mL of hot water at 95 ± 5 °C was prepared according to the brewing ratio of 1:1.5 (*w*/*v*), and then filtered using the same filtration procedure for all samples. All samples were treated identically during grinding, brewing, filtration, drying, storage, and subsequent oil extraction to minimize procedural variation. The CGs were then dried in an oven at 60 °C until a constant weight was achieved, after which they were stored at −20 °C for subsequent oil extraction.

### 2.3. Absorbance Measurement

Coffee roasting promotes Maillard reactions and the formation of brown-colored Maillard reaction products, especially melanoidins, which contribute to the color development of roasted coffee [13,14,15]. Therefore, absorbance at 420 nm was used as a relative indicator of browning intensity and Maillard reaction product formation. Specifically, 20 mg of each coffee ground sample was taken, extracted with methanol at a 1:10 (*w*/*v*) ratio, and the extracted solution was centrifuged at 3000 rpm for 10 min to obtain a clear supernatant. Then, 200 μL of the supernatant was placed into a 96-well plate, and absorbance was measured using an Epoch 2 microplate spectrophotometer (BioTek Instruments, Inc., Winooski, VT, USA). The measurements were performed in technical triplicate. Because several absorbance values approached or exceeded 1.0, the 420 nm values were used only as a relative index for comparing browning intensity among the present samples.

### 2.4. Extraction of Coffee Oil

50 g of dried CGs were soaked in 250 mL of n-hexane and extracted at room temperature under magnetic stirring at 200 rpm for 3 h, followed by filtration. The remaining residue was extracted twice more with 250 mL of n-hexane under the same temperature, duration, and agitation conditions. The two extracts were combined, washed three times with deionized water, and dehydrated using anhydrous sodium sulfate, allowing it to sit overnight. Finally, the solvent was removed using an EYELA N-1300 rotary evaporator (Tokyo Rikakikai Co., Ltd., Tokyo, Japan), and the oil samples were stored at −20 °C under nitrogen protection for further analysis.

### 2.5. Acid Value (AV)

10 g of coffee oil were added to 100 mL of an ethanol–ether mixture (1:1, *v*/*v*) and titrated with 0.1 M potassium hydroxide solution. Considering that coffee oil may contain caffeic acid and other weakly acidic phenolic compounds, 1% alkaline blue 6B-ethanol solution was used as the indicator to obtain a clearer titration endpoint in the ethanol–ether system. Alkali blue 6B has been reported as an alternative to phenolphthalein for acid value determination in oils containing phenolic minor components [16].

The acid value (AV) was calculated using the following formula [17]:

(1) AV = (A × f × 5.611)/S

where A is the volume of 0.1 M potassium hydroxide solution used for titration (mL), f is the factor of the solution, 5.611 is the equivalent free fatty acid content (mg) per 1 mL of 0.1 M potassium hydroxide solution, and S is the mass of the oil sample (g).

### 2.6. Peroxide Value (PV)

5 g of each oil sample was dissolved in an Erlenmeyer flask containing 50 mL of a solution of acetic acid and 2,2,4-trimethylpentane (3:2, *v*/*v*). To this mixture, 0.1 mL of saturated potassium iodide solution was added, and the mixture was shaken for 1 min. After adding 30 mL of purified water, the solution was titrated with 0.01 M sodium thiosulfate solution, using a 1% starch solution as the indicator. The peroxide value (PV) was calculated using the following equation [18]:

(2) PV (meq/kg) = (A × F × 10)/B

where A is the volume of 0.01 M sodium thiosulfate solution used for titration (mL), B is the mass of the oil sample (g), and F is the factor of the 0.01 M sodium thiosulfate solution used.

### 2.7. Fatty Acid Analysis

Fatty acid methyl esters were prepared by the boron trifluoride–methanol method [19]. 15 mg of each oil sample was added to a test tube with a glass stopper, followed by 1 mL of 0.5 mol/L NaOH-methanol solution. The mixture was heated in a water bath at 90 °C for 10 min for saponification. After the reaction was complete, 1 mL of 14% boron trifluoride-methanol solution was added, and the mixture was heated at 90 °C for 2 min for esterification. The reaction solution was then extracted with 5 mL of n-hexane, and impurities were removed by washing with saturated saline. Finally, the solution was dried with nitrogen, and the sample was dissolved in n-hexane to a 2% concentration for gas chromatography (GC) analysis.

The fatty acid methyl esters were analyzed by GC (GC-2010 PLUS, Shimadzu, Kyoto, Japan) equipped with a flame ionization detector [20]. The chromatographic conditions were as follows: a CP-Sil 88 capillary column (100 m × 0.25 mm, 0.25 μm, Agilent Technologies, Santa Clara, CA, USA), nitrogen as the carrier gas, and a flow rate of 1.0 mL/min. The temperature program was set as follows: initial temperature of 140 °C for 2 min, then increasing at a rate of 4 °C/min to 180 °C, followed by a further increase of 2 °C/min to 225 °C, which was maintained for 30 min. The injection volume was 1 μL with a split ratio of 1:50, and the injection port temperature was 240 °C, with the detector temperature set at 240 °C. The fatty acids were identified and quantified by comparing their retention times with those of standard fatty acids.

### 2.8. Triacylglycerol (TAG) Composition

The TAG composition of the oils was analyzed using a Waters 1525 HPLC system (Waters, Milford, MA, USA) equipped with a differential detector [21]. 500 mg of each oil sample were dissolved in acetone to a final volume of 10 mL, and 20 μL of the sample solution was injected into the HPLC system. The separation of TAG molecules was performed using a Develosil C30-UG-5 column (4.5 mm × 250 mm, Nomura Chemical Co., Ltd., Seto, Japan) with a mobile phase consisting of a 7:3 (*v*/*v*) mixture of acetone and acetonitrile. The flow rate was set at 1.0 mL/min, and the column temperature was maintained at 30 °C. Soybean oil was used as a standard to identify different types of TAG molecules.

### 2.9. Tocopherol

The tocopherol content in the oils was determined using a high-performance liquid chromatography (HPLC) system equipped with a UV detector (Agilent 1260, Agilent Technologies, Santa Clara, CA, USA) [22]. 1 g of each oil sample was dissolved in n-hexane to a final volume of 10 mL, and 10 μL was injected into the HPLC system. Separation of tocopherol isomers was achieved using a Shodex 5SIL-4E column (4.6 mm × 250 mm, Showa Denko K.K., Tokyo, Japan) with a mobile phase consisting of n-hexane and 2-propanol (999:3, *v*/*v*). The flow rate was set at 1.0 mL/min, and the column temperature was maintained at 40 °C. The UV detection wavelength was set at 298 nm. The content of tocopherol was calculated by comparing the peak areas of the samples with standard tocopherols (α-, β-, γ-, and δ-tocopherols).

### 2.10. Sterol Analysis

The sterol composition of the oils was analyzed by gas chromatography–mass spectrometry (GC-MS) [23]. 1 g of each oil sample was added to a test tube with a glass stopper, followed by 10 mL of 1 M potassium hydroxide-methanol solution and 1 mg of 5α-cholestan-3β-ol as an internal standard. The mixture was saponified at 90 °C for 1 h. After cooling to room temperature, 30 mL of warm water was added, and 20 mL of diethyl ether was used to extract the unsaponifiable matter three times. The combined ether phase was washed with 6 mL of water three times, dehydrated with anhydrous sodium sulfate, and allowed to sit overnight. The ether phase was recovered, concentrated using an EYELA N-1300 rotary evaporator (Tokyo Rikakikai Co., Ltd., Tokyo, Japan), and dissolved in chloroform to a concentration of 0.1 mg/mL, then stored at −20 °C for analysis.

GC-MS analysis was performed using a Shimadzu GC-MS-QP2010 Ultra system (Shimadzu, Kyoto, Japan). The chromatographic conditions were as follows: a DB-5 ms column (30 m × 0.25 mm, 0.25 μm, Agilent Technologies, Santa Clara, CA, USA), an injection port temperature of 300 °C, and a column temperature of 280 °C. Helium was used as the carrier gas, and the injection volume was 1 μL. The mass spectrometer was operated in electron impact mode with an ion source temperature of 230 °C, an interface temperature of 280 °C, and a scan range of m/z 20–440 with an event time of 0.4 s. Sterol components were identified by comparing their retention times and mass spectra with standard reference compounds and the NIST mass spectral library. Quantification was based on the ratio of the peak area of each target sterol to the internal standard peak area.

### 2.11. Folin-Reactive Compounds

Folin-reactive compounds were extracted from coffee oil using a solid-phase extraction method with a Sepax Generik Diol column (Sepax Technologies, Inc., Newark, DE, USA) [24]. 2 g of oil sample were dissolved in 5 mL of n-hexane. The column was activated with 6 mL of methanol and 6 mL of n-hexane. The oil sample dissolved in n-hexane was applied to the column, which was washed twice with 3 mL of n-hexane, followed by the addition of a 9:1 (*v*/*v*) n-hexane-ethyl acetate solution. The sample was eluted twice with 4 mL of methanol, and the eluate was collected in a 10 mL volumetric flask and brought to volume with methanol.

5 mL of the methanol eluate was mixed with 0.5 mL of Folin–Ciocalteu reagent for 3 min, followed by the addition of 1 mL of 7.5% sodium carbonate solution and a final volume of 10 mL with distilled water. The reaction mixture was allowed to stand for 2 h in the dark and then filtered to remove suspended particles and reduce turbidity before absorbance measurement. The absorbance was measured at 765 nm using an Epoch 2 microplate spectrophotometer (BioTek Instruments, Inc., Winooski, VT, USA). The content of total Folin-reactive compounds was determined by constructing a standard curve based on gallic acid gradient concentrations, and the results were expressed as gallic acid equivalents (mg GAE/kg). For the determination of total Folin-reactive compounds, the extraction and colorimetric assay were performed in technical triplicate for each oil sample. The relative standard deviations calculated from the triplicate measurements ranged from 1.5% to 5.2%, indicating acceptable analytical reproducibility. It should be noted that, during the oil extraction procedure described above, washing the hexane extract with deionized water may remove part of the polar phenolic or other reducing compounds. Therefore, the measured Folin-reactive compounds in the final oil samples may represent compounds retained in the oil phase after washing rather than the total Folin-reactive compounds originally present in the coffee grounds or crude hexane extract. In addition, because the Folin–Ciocalteu assay is based on a redox reaction and is not specific to phenolic compounds, the values obtained in this study should be interpreted as total Folin-reactive compounds rather than as the absolute content of individual phenolic compounds [25].

### 2.12. Statistical Analyses

All analytical measurements were performed in technical triplicate, and the results were expressed as mean ± standard deviation (Mean ± SD). Because the samples were commercial products rather than independently matched biological replicates, statistical differences should be interpreted as analytical differences within the present sample set rather than as definitive biological or causal effects of coffee species or roasting degree. Statistical analyses were performed using SPSS 25.0 software (IBM Corp., Armonk, NY, USA). Differences among groups were assessed by one-way analysis of variance (ANOVA), and Duncan’s multiple range test was used for post hoc comparisons. A significance level of *p* < 0.05 was considered statistically significant.

For multivariate analysis, all data were standardized using Z-score transformation prior to analysis. Principal Component Analysis (PCA) and Hierarchical Clustering Analysis (HCA) were conducted using R software (version 4.2.2). PCA was used to reduce dimensionality and identify distribution patterns based on lipid composition, while HCA (based on Euclidean distance and average linkage) was used to classify samples and variables. Visualization was performed through PCA score plots and heatmaps with dendrograms.

## 3. Results and Discussion

### 3.1. Oil Content

Table 2 presents oil contents of Arabica and Robusta CGs at various roasting degrees. In the Arabica sample group, oil contents tended to be higher in several roasted samples than in the unroasted sample. The unroasted Arabica sample (Au1) had an oil content of 12.13%, while the lightly roasted (Al2) and medium roasted (Am3) samples showed oil contents of 12.68% and 13.80%, respectively. The oil content reached its highest level of 15.14% in the deeply roasted (Ad6) sample. This suggests that higher roasting degree may be associated with increased oil extractability in some Arabica samples, possibly due to changes in coffee ground structure during roasting [26].

In contrast, the oil content of Robusta CGs was generally lower, and roasting had a less significant effect on oil release. The unroasted Robusta sample (Ru1) had an oil content of 10.10%, while the lightly roasted (Rl2) and medium roasted (Rm3) samples showed oil contents of 10.74% and 10.45%, respectively. Even in the deep roasting stages (Rd5 and Rd6), the oil contents exhibited only a moderate increase, reaching 10.26% and 11.42%, respectively. These results suggest that oil extractability differed among the selected Robusta samples, although this trend should be interpreted cautiously because sample origin and processing history were not fully controlled.

Overall, the selected Arabica CGs showed relatively higher oil contents than most Robusta samples. However, because the samples differed in origin, brand, and commercial processing history, the apparent differences in oil extractability should be interpreted cautiously [27].

This variation in oil release is not only closely related to the flavor and mouthfeel of coffee but may also influence their uses. Arabica coffee beans, known for their higher oil contents, typically exhibit a richer flavor and more pronounced aroma, making them suitable for premium coffee products such as specialty coffee, espresso, and pour-over brews. In contrast, Robusta coffee, with its lower oil content, is characterized by a more intense and robust taste. It is commonly used in instant coffee and low-cost coffee beverages; although its flavor is stronger, it lacks the complexity and richness of Arabica coffee [28].

### 3.2. Acid Value (AV) and Peroxide Value (PV)

Table 3 shows AV and PV of coffee oils at various roasting degrees. Both AV and PV were higher in the unroasted coffee oils, suggesting that roasted and unroasted samples differed in the chemical status of coffee bean lipids. In the unroasted samples, the AV of Arabica (Au1) was significantly higher than that of the roasted samples, which may be attributed to the presence of active lipases and the release of free fatty acids. During roasting, the breakdown of lipase activity and the heat-induced degradation of lipid structures reduced the AV. However, at the deep roasting stage, a slight increase in AV was observed, likely due to the formation of free fatty acids resulting from the cleavage of fatty acid chains during higher temperature roasting processes [29].

For PV, which measures the level of primary oxidation products (such as peroxides), roasted coffee oils exhibited significantly lower values than the unroasted oils. These findings may be related to the thermal instability of lipid hydroperoxides, which are primary oxidation products and can decompose during heat treatment. Therefore, lower PVs should not be directly interpreted as improved oxidative stability. The decomposition of hydroperoxides may generate secondary oxidation products, such as volatile aldehydes and ketones, which could affect the quality and flavor of coffee oils. Further analyses of secondary oxidation products are needed to evaluate the oxidative status and flavor stability of coffee oils more comprehensively [30,31].

### 3.3. Fatty Acid Composition

Fatty acid compositions of coffee oils are the crucial determinant of their physicochemical properties, nutritional value, and oxidative stability. Table 4 lists the major fatty acids in Arabica and Robusta coffee oils at various roasting degrees. The results show that the fatty acid profile of coffee oils is typical of vegetable oils, with the predominant fatty acids being palmitic acid (C16:0), stearic acid (C18:0), oleic acid (C18:1), and linoleic acid (C18:2). Palmitic acid is the major saturated fatty acid (SFA), accounting for 33.6% to 36.6% of the total fatty acids, while linoleic acid is the primary polyunsaturated fatty acid (PUFA), comprising 41.0% to 47.0%. Oleic acid, the main monounsaturated fatty acid (MUFA), fluctuated between 7.0% and 13.0%. Additionally, small amounts of stearic acid, linolenic acid (C18:3), and arachidic acid (C20:0) were also present.

The fatty acid composition of coffee oil is distinct from that of many other common edible oils, such as olive oil, canola oil, or sunflower oil, which typically have higher levels of oleic acid or linoleic acid. The “high palmitic acid + high linoleic acid + relatively low oleic acid” profile of coffee oil can serve as a distinctive chemical fingerprint, useful for identifying the source and authenticity of the oil. This profile also suggests that coffee oil may have unique applications in specific food industries or functional products that emphasize PUFA intake or require oils suitable for high-temperature processing.

In terms of species differences, Arabica coffee oils were richer in unsaturated fatty acids (UFAs), particularly PUFAs, compared to Robusta oils. For example, in the unroasted Arabica sample (Au1), linoleic acid accounted for 45.36%, and the total UFA content was 54.67%. In contrast, Robusta oils exhibited a higher proportion of MUFAs, especially in the deep-roasted Robusta sample (Rd5), where oleic acid content reached 13.22%, the highest among all the samples. This difference may reflect the combined influence of species background, geographical origin, and commercial processing history. In the present commercial sample set, Arabica oils tended to contain relatively higher PUFA levels, whereas some Robusta oils contained higher MUFA levels.

Differences in fatty acid compositions were also observed among samples with different roasting degrees. In general, contents of polyunsaturated fatty acids (such as linoleic acid and linolenic acid) slightly decreased with increasing roasting degree. This result contrasted with some previous reports, where PUFA content remained stable or even increased during roasting [32]. The reduction in PUFA content may be attributed to the chemical instability of double bonds in PUFAs, which are prone to oxidation and thermal degradation at high temperatures [33]. SFAs and some MUFAs (such as oleic acid), on the other hand, are more thermally stable and showed little to no loss during roasting.

### 3.4. sn-2 Fatty Acid Composition

The positional distribution of fatty acids in TAGs is an important factor in determining the nutritional value of oils [34]. Table 5 presents *sn*-2 fatty acid compositions in the twelve coffee oils with varying degrees of roasting. These included palmitic acid (C16:0), stearic acid (C18:0), oleic acid (C18:1), linoleic acid (C18:2), linolenic acid (C18:3), and arachidic acid (C20:0). The results showed that in all the samples, linoleic acid consistently dominates the *sn*-2 position, accounting for 65–78%, with an average value close to 70%, which is significantly higher than other fatty acids. Oleic acid and palmitic acid follow, with notable levels observed in certain samples. In general, the *sn*-2 position exhibited a clear enrichment in PUFAs, particularly linoleic acid.

In terms of varietal comparison, Arabica and Robusta coffee oils displayed distinct differences in the *sn*-2 fatty acid distribution. In Arabica samples, linoleic acid consistently predominates at the *sn*-2 position, ranging from 69.47% to 78.87%, with average values slightly higher than those of Robusta. Arabica samples, especially Al2 and Am3, exhibited the highest *sn*-2 linoleic acid content (up to 78.87%), indicating their stronger enrichment in polyunsaturated fatty acids. In contrast, Robusta samples tend to retain more monounsaturated and saturated fatty acids at the *sn*-2 position, showing a greater tendency toward fatty acid saturation. For instance, in sample Rd5, the *sn*-2 content of oleic acid and palmitic acid reached 15.97% and 11.67%, respectively. This structural difference suggests that the selected Robusta and Arabica coffee oils may have different lipid structural characteristics, but their thermal stability, oxidative resistance, and physiological effects require further direct evaluation.

Regarding the effect of roasting on *sn*-2 fatty acids, the overall trend is not pronounced. Although roasting may alter total fatty acid content—especially through thermal degradation of PUFAs—the fatty acid composition at the *sn*-2 position remains relatively stable. This indicated a degree of structural conservatism at the *sn*-2 position in the absence of significant lipid rearrangement or interesterification [35]. Similar phenomena have been reported in studies of plant oils, where the *sn*-2 position showed a selective enrichment in PUFAs and strong structural stability under thermal treatment [36].

### 3.5. TAG Composition

TAG is the predominant form of lipids in plant oils, consisting of one glycerol molecule esterified with three fatty acid chains. The fatty acid arrangement in TAGs influences melting point, oxidative stability, and absorption characteristics relevant to food processing [37,38]. In particular, the positional distribution of fatty acids on the TAG backbone (*sn*-1, *sn*-2, and *sn*-3) and the degree of saturation/unsaturation are the critical factors influencing structural characteristics and usages of the fats and oils.

Table 6 summarizes the major TAG species and their relative abundances in Arabica and Robusta coffee oils under different roasting levels. The most common TAG molecules in coffee oil include PLL (1-palmitoyl-2,3-linoleoyl-glycerol), PLP (1,3-dipalmitoyl-2-linoleoyl-glycerol), SLL (1-stearoyl-2,3-linoleoyl-glycerol), and OLL (1-oleoyl-2,3-linoleoyl-glycerol), reflecting the predominance of palmitic acid and linoleic acid in their fatty acid compositions. Among these, PLL and PLP are dominant across all the samples and play critical roles in determining the fluidity, melting behavior, and oxidative reactivity of coffee oils.

At the varietal level, although Arabica and Robusta coffee oils shared similar TAG profiles, they exhibited notable differences in the relative abundance of specific TAG species. Arabica coffee oil, with its higher linoleic acid content, is particularly rich in PLL, with significantly higher relative abundance than in Robusta. In contrast, Robusta coffee oil showed higher levels of PPL, reflecting a bias toward saturated and monounsaturated fatty acids. These structural differences stem from fundamental variations in their fatty acid compositions and are further manifested in their TAG assemblies. Notably, the PLL/PLP ratio may serve as a potential chemotaxonomic marker for distinguishing between coffee oil varieties. Arabica samples exhibited higher PLL content and greater TAG diversity, which may be associated with differences in oil physical properties. Conversely, Robusta samples contained relatively higher PLP levels, suggesting possible differences in lipid structural characteristics and processing properties.

In conjunction with the *sn*-2 fatty acid analysis discussed earlier, coffee oil is rich in TAG species such as PLL and PLP that contain PUFAs, which are generally present at much lower proportions in traditional vegetable oils such as olive oil and canola oil [39,40]. This structural feature confers unique functional potential to coffee oil. For example, PLL molecules containing PUFAs at the *sn*-2 position and saturated fatty acids at the *sn*-1/3 positions resemble the lipid structure of human milk fat, potentially enhancing lipid absorption and metabolic regulation in the human body [41]. Additionally, these highly unsaturated TAG structures contribute to the oil’s plasticity, making it particularly suitable for use in high-fat structural products such as chocolates, shortenings, and spreadable fats [42]. In our previous study, similar novel disaturated triacylglycerol-rich fats exhibited potential effects on improving chocolate qualities as these fats could blend with cocoa butter with desirable compatibilities [43,44].

Beyond PLL, Arabica coffee oils also contain higher levels of TAG species like LLL (1,2,3-linoleoyl-glycerol) and POL (1-palmitoyl-2-oleoyl-3-linoleoyl-glycerol), indicating greater unsaturation and structural flexibility. In contrast, Robusta is richer in moderately unsaturated TAGs such as POP and PPL, offering better thermal stability and greater molecular rigidity. These differences directly affect product mouthfeel and stability during processing and storage.

### 3.6. Polyphenol and Tocopherol Contents

Phenolic compounds and tocopherols are two important classes of antioxidant-related constituents found in plant oils. They are known not only for their capacities to scavenge reactive oxygen species and inhibit lipid peroxidation but also for their physiological functions such as delaying cellular aging, protecting vascular endothelial function, regulating cholesterol metabolism, and preventing various chronic cardiovascular diseases [45,46]. Table 7 presents the contents of total Folin-reactive compounds and various tocopherol isomers (α-, β-, γ-, and δ-) in Arabica and Robusta coffee oil samples. These constituents are relevant to the compositional characteristics of coffee oils and may provide useful information for their potential use in food additives, nutraceuticals, and cosmetics.

Overall, the content of total Folin-reactive compounds did not differ markedly between Arabica and Robusta coffee oils, but substantial variation was observed among samples with different roasting degrees. This reflected from a combined effect of varietal genetics, geographical origin, and roasting conditions. Unroasted samples exhibited relatively low polyphenol contents—55.91 mg/100 g (Au1) and 49.50 mg/100 g (Ru1)—whereas deeply roasted samples, such as Ad5 and Rd5, showed significantly elevated polyphenol levels at 189.85 mg/100 g and 205.40 mg/100 g, respectively. This trend suggested that roasting may influence the levels of Folin-reactive compounds in coffee oils, possibly through cell wall degradation and the release or transformation of reducing compounds. Phenolic compounds in coffee beans, such as chlorogenic acid and caffeic acid, may exist in bound or encapsulated forms within the cell wall and polysaccharide matrix in green beans. During brewing, many water-soluble phenolic compounds may be extracted into the brew. However, high roasting temperatures may disrupt these cellular structures, enabling some phenolic or other reducing compounds to migrate into the oil phase. In addition, thermal transformation, polymerization, and condensation reactions during roasting may generate compounds that react with the Folin–Ciocalteu reagent, thereby increasing the measured Folin-reactive compound content [47]. These findings indicated that, although many water-soluble phenolic compounds may be extracted during brewing, roasting may still affect the levels of Folin-reactive compounds in coffee oil, providing compositional information for its further evaluation as a functional lipid ingredient.

Regarding tocopherols, the levels of coffee oils ranged from 651.67 to 1495.37 mg/kg, which were significantly higher than most commercial oils and novel special oils [48,49,50]. For more details, α-, β-, and γ-isomers were detected in both Arabica and Robusta samples, whereas δ-tocopherol was not found in any sample, likely due to its naturally low abundance or thermal sensitivity. The total tocopherol content varied significantly by variety and roasting level: Arabica samples consistently exhibited higher tocopherol levels than Robusta, with certain samples (e.g., Ad6 and Am3) reaching 1453.76–1495.37 mg/kg, more than twice the levels found in deeply roasted Robusta samples (e.g., Rd6). This indicated a strong varietal influence on tocopherol accumulation capacity. Notably, β-tocopherol emerged as the predominant isomer in coffee oil, which differs markedly from conventional plant oils such as soybean, corn, and sunflower oils that are typically rich in α- and γ-tocopherols [51]. For example, in the Ad6 sample, β-tocopherol reached as high as 1055.37 mg/kg, accounting for over 70% of the total tocopherols.

Although coffee oil is naturally rich in tocopherols, high-temperature roasting inevitably causes some degree of degradation, particularly for α- and γ-tocopherols, which are prone to thermal oxidation and volatilization. Data showed a general decline in these tocopherols with increasing roast intensity, suggesting lower thermal stability. β-Tocopherol, due to its relatively stable molecular structure, degrades more slowly but also showed losses under deep roasting (e.g., Rd6). This highlighted the requirements to optimize roasting conditions in practical applications. Even after roasting, all the coffee oils maintained relatively abundant tocopherol levels. In particular, their tocopherol levels may support further investigation of coffee oil as a tocopherol-rich ingredient for fortified foods and related products.

### 3.7. Squalene and Phytosterol Profiles

In plant oils, aside from fatty acids and phenolic antioxidants, squalene and phytosterols, as key components of the unsaponifiable fraction, also play multiple biological roles in maintaining human health. These compounds are involved not only in stabilizing cell membrane structures but also in regulating blood lipids, inhibiting cholesterol absorption, and exhibiting anti-inflammatory, antioxidant, antitumor, and immunomodulatory activities [52,53]. As a non-traditional plant oil with a unique botanical origin, the squalene and sterol profile of coffee oil is crucial for evaluating its nutritional values and multifunctional development potential.

Squalene is a 30-carbon triterpene non-polar compound, widely found in deep-sea shark liver oil, olive oil, and human sebaceous secretions. It serves as a vital biosynthetic precursor for sterols and steroids. Due to its antioxidant and UV-protective properties, squalene is widely used in skincare and medical lipid formulations [54,55]. Results from Table 7 revealed significant varietal differences in squalene content among coffee oil samples. For example, sample Au1 contained only 15.49 mg/100 g, while Am3 was slightly higher at 27.64 mg/100 g. In contrast, Robusta samples showed markedly higher squalene levels, with the deeply roasted Rd6 reaching 170.37 mg/100 g, more than six times the highest value observed in Arabica. Robusta’s higher squalene levels may result from enhanced triterpene synthesis under tropical stress conditions. Furthermore, squalene’s stable molecular structure makes it resistant to thermal degradation and oxidation, enabling its effective enrichment in the lipid phase even under moderate roasting. This property supports its potential application in medical skincare, antioxidant lipid matrices, and beauty-focused nutraceuticals.

Regarding phytosterols, five major types were detected in coffee oil: campesterol, stigmasterol, β-sitosterol, Δ^7^-stigmastenol, and Δ^7^-avenasterol, with β-sitosterol being the predominant sterol. Overall, Robusta coffee oil exhibited significantly higher total sterol content than Arabica. For instance, total sterol content in Arabica sample Au1 was 467.97 mg/100 g, whereas Robusta samples Rd5 and Rd6 reached 1007.92 mg/100 g and 863.51 mg/100 g, nearly double that of Arabica. In particular, the β-sitosterol content in Robusta sample Rd5 was as high as 497.40 mg/100 g, approximately 2.5 times that of Au1.

β-sitosterol is recognized for its ability to competitively inhibit cholesterol absorption by binding to intestinal cholesterol transporters such as NPC1L1, making it a widely accepted bioactive ingredient in cholesterol-lowering functional foods [56]. Its high concentration in coffee oil supports its potential in cardiovascular health interventions. Of special scientific interest is the presence of relatively high levels of Δ^7^-sterols (Δ^7^-stigmastenol and Δ^7^-avenasterol), which are rarely found in traditional plant oils like olive or corn oil. These Δ^7^-sterols have been shown to exhibit stronger membrane-stabilizing, antioxidant, and anticancer activities [57,58]. Their presence enhances the unique nutritional and functional value of coffee oil, especially in the context of high-end nutrition and bioactive lipid product development. For instance, in Robusta sample Ru1, Δ^7^-avenasterol content reached 206.42 mg/100 g, compared to only 46.70 mg/100 g in Arabica sample Am4, indicating significant varietal differences.

Unlike thermolabile compounds such as tocopherols, both squalene and phytosterols demonstrate strong thermal stability. The results showed that sterol content and composition remained relatively consistent between medium and dark roasted samples, indicating structural integrity under thermal stress. This stability suggests that the unsaponifiable components in coffee oils retain their physiological functionality even after high-temperature processing, supporting their wide applicability in heat-processed foods, baked goods, and skincare formulations.

### 3.8. Chemometric Analysis

**Figure 2:**
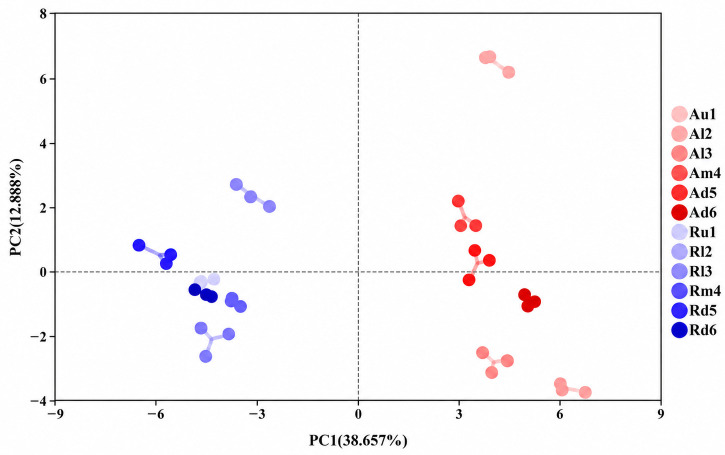
Principal component analysis score plot of coffee oils based on lipid compositions.

In this study, a multivariate chemometric approach was employed to systematically analyze 12 coffee oil samples derived from different roasting levels and coffee varieties. Principal component analysis (PCA) revealed that the first two principal components (PC1 and PC2) explained 51.6% of the total variance, clearly distinguishing Arabica and Robusta samples based on their distribution patterns (Figure 2). Arabica samples exhibited consistently higher scores along the PC1 axis, while Robusta samples clustered predominantly in the lower PC1 region. This indicated that, in the present commercial sample set, the overall lipid profiles were more closely associated with the Arabica and Robusta sample groups than with the assigned roasting levels.

**Figure 3:**
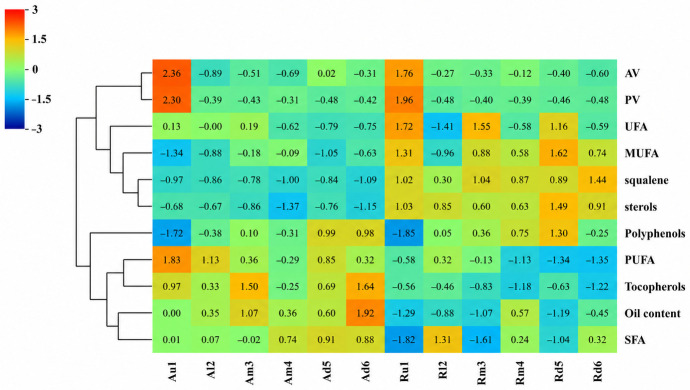
Heatmap and hierarchical clustering analysis of lipid components in coffee oils.

Hierarchical cluster analysis (HCA) and heatmap results (Figure 3) further supported the classification trend observed in PCA. The inter-varietal differences were particularly evident in the content of selected minor lipid components. Arabica coffee oils showed relatively higher levels of Folin-reactive compounds and tocopherols, whereas Robusta samples had higher proportions of oleic acid, squalene, and phytosterols. These compositional differences may be related to coffee species, but they may also be influenced by geographical origin, brand, and commercial processing history.

Roasting-related variations were also observed in the compositions of coffee oils. Specifically, roasted samples showed lower acid and peroxide values, while the levels of lipids, Folin-reactive compounds, and tocopherols varied among the samples. This trend was more evident in some dark-roasted samples, which showed relatively higher levels of selected minor components.

## 4. Conclusions

This study systematically analyzed the lipid composition and selected minor components of coffee oils extracted from Arabica and Robusta spent CGs under different roasting conditions. In the present commercial sample set, Arabica and Robusta coffee oils showed distinct compositional characteristics, with Arabica samples generally containing higher levels of tocopherols and unsaturated fatty acids, whereas Robusta samples contained higher levels of squalene and sterols.

In summary, dark-roasted Arabica coffee oil showed compositional features associated with relatively high tocopherol and Folin-reactive compound levels, while Robusta coffee oil showed compositional features characterized by higher levels of squalene, phytosterols, and relatively saturated lipid structures. These findings provide compositional information for the targeted and sustainable utilization of coffee oils from selected commercial coffee ground samples.

## Figures and Tables

**Table 1:** Species, brand, origin, and roast degree of coffee beans.

Species	Brand	Origin of Coffee Beans	Roast Degree	Absorbance at 420 nm	Abbreviation
Arabica	Starhouse	El Salvador	Unroasted	0.147 ± 0.002	Au1
	Illy	Ethiopia	Light	0.503 ± 0.002	Al2
	Starbucks	Malaysia	Medium	0.992 ± 0.008	Am3
	Sinloy	China	Medium	0.864 ± 0.006	Am4
	Starbucks	Malaysia	Dark	1.326 ± 0.009	Ad5
	Sinloy	China	Dark	1.380 ± 0.003	Ad6
Robusta	Leka	Uganda	Unroasted	0.165 ± 0.003	Ru1
	Toba blue-eye	Indonesia	Light	0.591 ± 0.002	Rl2
	Xingke	China	Medium	0.740 ± 0.013	Rm3
	G7	Vietnam	Medium	0.925 ± 0.002	Rm4
	Caffe Borbone	Indonesia	Dark	1.186 ± 0.012	Rd5
	Sinloy	India	Dark	1.268 ± 0.008	Rd6

Values represent the means ± SD from technical triplicate measurements.

**Table 2:** Oil contents of coffee grounds with different varieties and roasting levels.

	Arabica						Robusta					
	Au1	Al2	Am3	Am4	Ad5	Ad6	Ru1	Rl2	Rm3	Rm4	Rd5	Rd6
Oil content (%)	12.13 ± 0.34 d	12.68 ± 0.15 c	13.80 ± 0.09 b	12.69 ± 0.15 c	13.07 ± 0.11 c	15.14 ± 0.24 a	10.10 ± 0.26 g	10.74 ± 0.34 f	10.45 ± 0.25 fg	13.01 ± 0.12 c	10.26 ± 0.39 g	11.42 ± 0.31 e

Values represent the means ± SD from technical triplicate measurements. In each row, different letters mean significant differences (*p* ≤ 0.05).

**Table 3:** Acid values and peroxide values of coffee oils with different varieties and roasting levels.

	Arabica						Robusta					
	Au1	Al2	Am3	Am4	Ad5	Ad6	Ru1	Rl2	Rm3	Rm4	Rd5	Rd6
AV (mgKOH/g)	8.49± 0.13 a	3.21± 0.05 j	3.83± 0.06 g	3.54± 0.05 i	4.69± 0.07 c	4.15± 0.06 e	7.51± 0.11 b	4.22± 0.06 e	4.12± 0.06 ef	4.46± 0.07 d	4.01± 0.06 f	3.68± 0.06 h
PV (meq/kg)	3.13± 0.10 a	0.54± 0.04 c	0.50± 0.03 cd	0.62± 0.03 b	0.45± 0.03 d	0.51± 0.03 cd	2.80±0.05 a	0.45± 0.02 d	0.53± 0.03 c	0.54± 0.04 c	0.47± 0.02 cd	0.45± 0.03 d

Values represent the means ± SD from technical triplicate measurements. In each row, different letters mean significant differences (*p* ≤ 0.05).

**Table 4:** Fatty acid compositions of coffee oils with different varieties and roasting levels.

	Arabica						Robusta					
Fatty Acid (%)	Au1	Al2	Am3	Am4	Ad5	Ad6	Ru1	Rl2	Rm3	Rm4	Rd5	Rd6
C12:0	0.01 ± 0.00 c	0.02 ± 0.00 c	0.01 ± 0.00 c	0.02 ± 0.00 c	0.02 ± 0.00 c	0.01 ± 0.00 c	0.02 ±0.00 c	0.04 ± 0.00 b	0.01 ± 0.00 c	0.05 ± 0.01 a	0.03 ± 0.02 bc	0.04 ± 0.01 b
C14:0	0.06 ± 0.00 ab	0.07 ± 0.00 ab	0.05 ± 0.01 b	0.06 ± 0.00 ab	0.06 ± 0.01 ab	0.06 ± 0.00 ab	0.08 ± 0.01 ab	0.10 ± 0.00 a	0.05 ± 0.04 b	0.08 ± 0.01 ab	0.08 ± 0.01 ab	0.08 ± 0.01 ab
C16:0	36.57 ± 0.23 a	33.61 ± 0.38 d	35.07 ± 0.08 c	34.92 ± 0.16 c	35.30 ± 0.25 bc	36.25 ± 0.07 a	33.33 ± 0.11 d	34.76 ± 0.19 c	33.20 ± 0.22 d	35.40 ± 0.08 bc	33.44 ± 0.04 d	35.53 ± 0.25 b
C16:1	0.09 ± 0.00 a	0.09 ± 0.00 a	0.05 ± 0.04 b	0.09 ± 0.00 a	0.08 ± 0.00 ab	0.08 ± 0.00 ab	0.06 ± 0.00 b	0.05 ± 0.00 b	0.06 ± 0.01 b	0.07 ± 0.01 ab	0.07 ± 0.00 ab	0.06 ± 0.00 b
C18:0	5.70 ±0.03 h	7.61 ± 0.11 a	6.63 ± 0.02 ef	7.41 ± 0.05 b	7.16 ± 0.08 c	6.52 ± 0.03 f	6.70 ± 0.02 e	7.00 ± 0.06 d	6.51 ± 0.14 f	6.37 ± 0.00 g	6.98 ± 0.12 d	6.62 ± 0.09 ef
C18:1	7.37 ± 0.04 l	8.25 ± 0.04 i	9.68 ± 0.06 g	9.80 ± 0.07 f	7.94 ± 0.03 k	8.77 ± 0.01 h	12.61 ± 0.02 b	8.10 ± 0.04 j	11.75 ± 0.03 c	11.16 ± 0.04 e	13.22 ± 0.09 a	11.48 ± 0.04 d
C18:2	45.36 ± 0.31 a	44.66 ± 0.10 b	43.32 ± 0.12 e	42.48 ± 0.34 f	44.02 ± 0.09 c	43.71 ± 0.08 d	42.39 ± 0.04 f	43.85 ± 0.08 d	43.02 ± 0.11 e	41.37 ± 0.08 g	41.08 ± 0.01 h	41.40 ± 0.09 g
C18:3	1.71 ± 0.04 a	1.35 ± 0.09 c	1.52 ± 0.04 b	1.38 ± 0.04 c	1.57 ± 0.07 b	1.07 ± 0.19 e	1.03 ± 0.01 e	0.93 ± 0.10 e	1.07 ± 0.02 e	1.19 ± 0.03 d	1.17 ± 0.05 d	0.82 ± 0.01 f
C20:0	1.95 ± 0.01 h	2.85 ± 0.11 c	2.39 ± 0.04 f	2.48 ± 0.03 e	2.47 ± 0.06 e	2.34 ± 0.02 f	2.51 ± 0.01 e	3.59 ± 0.07 a	2.93 ± 0.02 b	2.64 ± 0.03 d	2.64 ± 0.03 d	2.25 ± 0.03 g
C20:1	0.04 ± 0.01 c	0.06 ± 0.01 b	0.06 ± 0.00 b	0.05 ± 0.01 b	0.06 ± 0.01 b	0.06 ± 0.00 b	0.06 ± 0.00 b	0.08 ± 0.01 a	0.08 ± 0.01 a	0.06 ± 0.00 b	0.06 ± 0.00 b	0.07 ± 0.00 b
C20:2	0.05 ± 0.00 a	0.04 ± 0.00 ab	0.03 ± 0.02 b	0.03 ± 0.00 b	0.04 ± 0.00 ab	0.04 ± 0.00 ab	0.03 ± 0.02 b	0.04 ± 0.00 ab	0.04 ± 0.02 ab	0.05 ± 0.01 a	0.04 ± 0.01 ab	0.05 ± 0.01 a
C22:0	0.46 ± 0.01 f	0.72 ± 0.03 a	0.51 ± 0.01 e	0.56 ± 0.01 c	0.61 ± 0.02 b	0.43 ± 0.00 f	0.37 ± 0.01 g	0.53 ± 0.01 d	0.44 ± 0.01 f	0.43 ± 0.00 f	0.44 ± 0.02 f	0.49 ± 0.00 e
C22:1	0.07 ± 0.01 ab	0.08 ± 0.01 ab	0.08 ± 0.00 ab	0.09 ± 0.03 a	0.07 ± 0.00 ab	0.06 ± 0.00 b	0.07 ± 0.00 ab	0.09 ± 0.00 a	0.06 ± 0.03 b	0.06 ± 0.01 b	0.07 ± 0.01 ab	0.07 ± 0.00 ab
C24:0	0.15 ± 0.09 c	0.09 ± 0.10 d	0.23 ± 0.01 b	0.21 ± 0.01 b	0.21 ± 0.01 b	0.20 ± 0.00 b	0.21 ± 0.01 b	0.24 ± 0.03 a	0.22 ± 0.01 b	0.18 ± 0.03 b	0.21 ± 0.05 b	0.21 ± 0.00 b
Other	0.42 ± 0.22 b	0.50 ± 0.21 b	0.39 ± 0.14 b	0.41 ± 0.14 b	0.39 ± 0.19 b	0.39 ± 0.13 b	0.54 ± 0.21 b	0.60 ± 0.07 ab	0.56 ± 0.15 b	0.89 ± 0.01 a	0.49 ± 0.07 b	0.82 ± 0.16 a
∑SFA	44.91 ± 0.16 d	44.97 ± 0.23 cd	44.88 ± 0.05 d	45.67 ± 0.22 b	45.84 ± 0.09 b	45.81 ± 0.12 b	43.01 ± 0.25 f	46.26 ± 0.11 a	43.23 ± 0.18 f	45.15 ± 0.11 cd	43.82 ± 0.17 e	45.23 ± 0.12 c
∑MUFA	7.56 ± 0.04 l	8.48 ± 0.04 i	9.86 ± 0.10 g	10.03 ± 0.04 f	8.14 ± 0.03 k	8.97 ± 0.01 h	12.80 ± 0.03 b	8.32 ± 0.04 j	11.95 ± 0.04 c	11.35 ± 0.05 e	13.41 ± 0.09 a	11.68 ± 0.04 d
∑PUFA	47.11 ± 0.35 a	46.05 ± 0.07 b	44.87 ± 0.07 d	43.89 ± 0.33 e	45.62 ± 0.14 c	44.82 ± 0.25 d	43.45 ± 0.02 f	44.82 ± 0.10 d	44.13 ± 0.13 e	42.61 ± 0.07 g	42.28 ± 0.07 h	42.27 ± 0.08 h
∑UFA	54.67 ± 0.38 c	54.54 ± 0.06 c	54.73 ± 0.15 c	53.92 ± 0.36 d	53.76 ± 0.16 d	53.80 ± 0.25 d	56.25 ± 0.05 a	53.14 ± 0.14 e	56.08 ± 0.10 a	53.96 ± 0.11 d	55.69 ± 0.11 b	53.95 ± 0.11 d

SFA, saturated fatty acid; UFA, unsaturated fatty acid; MUFA, monounsaturated fatty acid; PUFA, polyunsaturated fatty acid. Values represent the means ± SD from technical triplicate measurements. In each row, different letters mean significant differences (*p* ≤ 0.05).

**Table 5:** *sn*-2 Fatty acid compositions of coffee oils with different varieties and roasting levels.

	Arabica						Robusta					
Fatty Acid (%)	Au1	Al2	Am3	Am4	Ad5	Ad6	Ru1	Rl2	Rm3	Rm4	Rd5	Rd6
C16:0	6.93 ± 0.13 g	8.28 ± 0.07 f	6.45 ± 0.36 h	12.45 ± 0.30 a	11.19 ± 0.20 c	8.67 ± 0.28 e	9.41 ± 0.02 d	12.58 ± 0.04 a	9.59 ± 0.03 d	11.00 ± 0.20 c	11.67 ± 0.16 b	9.57 ± 0.19 d
C18:0	4.08 ± 0.12 a	1.70 ± 0.16 d	1.72 ± 0.47 d	3.21 ± 0.24 b	2.55 ± 0.09 c	1.93 ± 0.04 d	1.78 ± 0.30 d	3.29 ± 0.05 b	1.87 ± 0.19 d	2.77 ± 0.19 c	3.44 ± 0.37 b	3.23 ± 0.08 b
C18:1	12.69 ± 0.44 b	9.83 ± 0.50 f	10.73 ± 0.08 e	12.92 ± 0.20 b	11.28 ± 0.26 d	12.75 ± 0.26 b	12.74 ± 0.39 b	12.74 ± 0.14 b	12.59 ± 0.02 bc	12.13 ± 0.30 c	15.97 ± 0.34 a	13.24 ± 0.31 b
C18:2	74.63 ± 0.86 b	77.86 ± 0.61 a	78.87 ± 0.76 a	69.47 ± 0.73 e	73.38 ± 0.68 c	74.56 ± 0.61 b	74.76 ± 0.45 b	70.53 ± 0.14 e	74.86 ± 0.22 b	72.68 ± 0.98 cd	67.87 ± 0.50 f	72.12 ± 0.60 d
C18:3	1.42 ± 0.14 c	2.24 ± 0.16 a	2.14 ± 0.15 a	1.83 ± 0.02 b	1.43 ± 0.11 c	1.96 ± 0.06 ab	1.14 ± 0.18 de	0.54 ± 0.00 f	0.95 ± 0.02 e	1.20 ± 0.21 d	0.63 ± 0.04 f	1.52 ± 0.03 c
C20:0	0.25 ± 0.02 bc	0.09 ± 0.03 d	0.10 ± 0.01 d	0.13 ± 0.01 d	0.18 ± 0.03 c	0.14 ± 0.02 d	0.15 ± 0.02 d	0.32 ± 0.09 b	0.15 ± 0.06 d	0.21 ± 0.08 c	0.42 ± 0.05 a	0.32 ± 0.01 b

Values represent the means ± SD from technical triplicate measurements. In each row, different letters mean significant differences (*p* ≤ 0.05).

**Table 6:** Triacylglycerol compositions of coffee oils with different varieties and roasting levels.

		Arabica						Robusta					
ECN	TAG (%)	Au1	Al2	Am3	Am4	Ad5	Ad6	Ru1	Rl2	Rm3	Rm4	Rd5	Rd6
40	LLLn	1.71 ± 0.04 b	1.78 ± 0.06 b	1.32 ± 0.05 c	2.22 ± 0.08 a	2.34 ± 0.09 a	2.34 ± 0.08 a	1.34 ± 0.02 c	1.37 ± 0.08 c	1.07 ± 0.09 e	1.22 ± 0.06 cd	1.35 ± 0.11 c	1.11 ± 0.07 e
42	LLL	5.33 ± 0.09 b	6.24 ± 0.13 a	5.99 ± 0.47 a	5.61 ± 0.25 b	5.91 ± 0.22 ab	5.67 ± 0.10 b	5.63 ± 0.40 b	5.35 ± 0.30 b	6.07 ± 0.45 a	5.13 ± 0.26 b	6.04 ± 0.55 a	5.35 ± 0.26 b
	OLLn	0.33 ± 0.02 ab	0.21 ± 0.02 d	0.29 ± 0.02 b	0.31 ± 0.01 b	0.34 ± 0.02 a	0.30 ± 0.03 b	0.20 ± 0.01 d	0.25 ± 0.02 c	0.21 ± 0.02 d	0.25 ± 0.01 c	0.19 ± 0.02 d	0.31 ± 0.02 b
	PLLn	1.77 ± 0.08 b	2.14 ± 0.03 a	1.79 ± 0.04 b	1.86 ± 0.02 b	1.12 ± 0.05 d	1.83 ± 0.07 b	1.11 ± 0.08 d	0.90 ± 0.05 e	1.18 ± 0.10 d	1.33 ± 0.07 c	1.39 ± 0.11 c	1.20 ± 0.07 d
44	OLL	4.70 ± 0.09 c	4.19 ± 0.09 c	5.88 ± 0.18 a	4.70 ± 0.17 c	4.44 ± 0.15 c	4.85 ± 0.10 b	5.22 ± 0.37 b	4.40 ± 0.25 c	4.12 ± 0.27 c	4.72 ± 0.25 bc	4.57 ± 0.18 c	4.75 ± 0.34 bc
	PLL	32.80 ± 1.76 a	33.04 ± 1.06 a	28.95 ± 2.11 b	29.67 ± 1.26 b	29.64 ± 2.05 b	29.22 ± 1.65 b	26.14 ± 1.72 bc	27.15 ± 1.52 bc	25.09 ± 2.15 c	26.43 ± 0.82 bc	25.76 ± 1.95 c	24.75 ± 0.41 c
46	OOL	1.91 ± 0.08 d	1.92 ± 0.06 d	3.02 ± 0.09 b	2.82 ± 0.21 bc	2.64 ± 0.18 c	2.44 ± 0.18 c	3.41 ± 0.24 a	2.48 ± 0.12 c	3.47 ± 0.29 a	2.49 ± 0.13 c	2.46 ± 0.20 c	2.88 ± 0.14 bc
	PLnS	0.52 ± 0.05 e	0.91 ± 0.03 a	0.45 ± 0.03 f	0.70 ± 0.04 c	0.63 ± 0.04 d	0.81 ± 0.03 b	0.43 ± 0.03 fg	0.25 ± 0.02 h	0.40 ± 0.03 g	0.36 ± 0.02 g	0.38 ± 0.03 g	0.37 ± 0.02 g
	SLL	11.28 ± 0.84 c	10.67 ± 0.96 c	12.94 ± 0.60 b	13.01 ± 0.34 b	13.21 ± 0.60 b	13.03 ± 0.53 b	14.97 ± 0.49 ab	14.41 ± 0.80 ab	16.41 ± 1.25 a	15.92 ± 0.59 a	15.17 ± 1.05 ab	15.91 ± 0.97 a
	POL	6.73 ± 0.09 a	4.57 ± 0.39 bc	5.57 ± 0.45 b	5.76 ± 0.22 b	4.99 ± 0.43 bc	5.56 ± 0.08 b	4.84 ± 0.34 bc	5.04 ± 0.28 bc	4.56 ± 0.39 bc	4.35 ± 0.23 c	5.40 ± 0.35 b	4.79 ± 0.34 bc
	PLP	28.84 ± 0.80 b	31.57 ± 0.89 ab	29.65 ± 1.75 ab	29.36 ± 1.71 b	30.76 ± 2.14 ab	29.85 ± 1.26 ab	31.52 ± 1.46 ab	33.70 ± 1.86 a	31.61 ± 2.51 ab	32.58 ± 1.06 ab	31.77 ± 1.89 ab	33.24 ± 1.24 a
48	PLS	1.47 ± 0.06 d	1.23 ± 0.10 e	1.75 ± 0.17 cd	1.56 ± 0.04 cd	1.70 ± 0.14 cd	1.88 ± 0.10 c	2.54 ± 0.18 ab	2.19 ± 0.12 b	2.76 ± 0.24 a	2.35 ± 0.12 ab	2.21 ± 0.17 b	2.19 ± 0.13 b
	PPP	2.60 ± 0.17 ab	1.54 ± 0.18 c	2.38 ± 0.14 ab	2.43 ± 0.16 ab	2.27 ± 0.18 ab	2.23 ± 0.12 b	3.26 ± 0.23 a	2.51 ± 0.14 ab	3.05 ± 0.26 a	2.86 ± 0.15 ab	3.32 ± 0.26 a	3.14 ± 0.19 a

ECN, equivalent carbon number; P, 16:0; S, 18:0; O, 18:1; L, 18:2; Ln, 18:3. The total triacylglycerol measured normalized to 100%. Values represent the means ± SD from technical triplicate measurements. In each row, different letters mean significant differences (*p* ≤ 0.05).

**Table 7:** Total Folin-reactive compounds, tocopherols, squalene and sterols of coffee oils with different varieties and roasting levels.

		Arabica						Robusta					
Category	Subclass	Au1	Al2	Am3	Am4	Ad5	Ad6	Ru1	Rl2	Rm3	Rm4	Rd5	Rd6
Total Folin-reactive compounds (mg GAE/kg)		55.91 ± 2.05 g	122.01 ± 4.00 f	145.83 ± 2.52 e	125.49 ± 5.05 f	189.85 ± 7.28 b	189.38 ± 3.75 b	49.50 ± 0.97 g	143.26 ± 2.17 e	158.70 ± 8.18 d	178.02 ± 8.56 c	205.40 ± 6.35 a	128.82 ± 2.96 f
Tocopherols (mg/kg)	α- tocopherols	273.64 ± 8.39 e	414.62 ± 7.30 b	723.12 ± 9.97 a	220.76 ± 4.37 fg	395.32 ± 7.76 c	391.68 ± 6.15 c	202.31 ± 4.63 h	349.78 ± 9.44 d	202.24 ± 8.79 h	231.19 ± 6.80 f	264.62 ± 8.96 e	217.42 ± 7.68 g
	β- tocopherols	1007.33 ± 16.93 b	655.00 ± 10.07 e	669.37 ± 12.38 de	676.08 ± 11.93 d	792.10 ± 10.42 c	1055.37 ± 10.35 a	619.12 ± 9.69 f	471.22 ± 13.44 i	524.76 ± 2.93 h	420.48 ± 5.94 j	552.49 ± 9.98 g	386.41 ± 7.18 k
	γ- tocopherols	13.93 ± 0.25 h	34.66 ± 0.77 d	61.27 ± 1.31 a	33.51 ± 0.52 d	24.13 ± 0.53 f	48.32 ± 1.54 b	16.21 ± 0.68 g	46.21 ± 1.09 c	28.37 ± 0.49 e	Tr.	Tr.	33.85 ± 0.83 d
	δ- tocopherols	N. D	N. D	N. D	N. D	N. D	N. D	N. D	N. D	N. D	N. D	N. D	N. D
	Total	1294.90 ± 11.66 c	1104.28 ± 9.04 e	1453.76 ± 11.76 b	930.35 ± 9.34 f	1211.55 ± 7.22 d	1495.37 ± 13.63 a	837.64 ± 5.82 h	867.21 ± 5.52 g	755.37 ± 8.88 j	651.67 ± 3.41 k	817.11 ± 2.97 i	637.69 ± 3.18 k
Squalene (mg/100 g)		15.49 ± 0.18 g	22.13 ± 0.44 f	27.64 ± 0.82 e	13.04 ± 0.68 g	23.35 ± 0.71 f	7.61 ± 0.46 h	143.44 ± 2.48 b	97.00 ± 1.98 d	144.47 ± 2.94 b	133.51 ± 2.05 c	135.06 ± 4.16 c	170.37 ± 3.47 a
Sterols (mg/100 g)	campesterol	60.68 ± 2.60 h	81.97 ± 2.99 f	61.73 ± 2.87 h	33.17 ± 2.04 j	77.10 ± 2.34 g	44.06 ± 1.54 i	137.44 ± 2.93 c	128.54 ± 3.71 d	166.73 ± 2.67 b	136.50 ± 2.56 c	199.13 ± 2.96 a	117.26 ± 1.16 e
	stigmasterol	77.61 ± 3.56 g	101.72 ± 3.79 e	65.47 ± 3.16 h	47.74 ± 0.48 i	95.31 ± 1.41 f	63.51 ± 1.20 h	129.25 ± 5.66 d	145.29 ± 5.82 c	174.04 ± 4.88 b	135.37 ± 3.29 d	180.52 ± 1.77 a	131.82 ± 2.77 d
	β-sitosterol	200.73 ± 4.12 g	183.01 ± 5.51 h	203.32 ± 6.01 g	134.65 ± 2.96 j	179.67 ± 5.45 h	151.65 ± 3.16 i	374.87 ± 8.32 c	360.59 ± 7.11 d	270.16 ± 8.17 f	346.91 ± 6.29 e	497.40 ± 8.22 a	441.13 ± 9.87 b
	△7 stigmastenol	65.82 ± 3.31 a	22.93 ± 1.24 d	18.88 ± 0.56 e	36.08 ± 1.47 c	21.02 ± 0.64 de	36.32 ± 0.97 c	47.51 ± 1.01 b	48.56 ± 1.94 b	36.67 ± 1.75 c	35.34 ± 1.24 c	34.09 ± 1.25 c	21.31 ± 1.10 de
	△7 avenasterol	63.13 ± 2.53 h	81.08 ± 2.94 f	74.95 ± 4.09 g	46.70 ± 1.24 j	75.91 ± 1.60 g	56.33 ± 1.03 i	206.42 ± 1.79 a	165.56 ± 3.25 b	139.70 ± 0.81 d	139.74 ± 2.48 d	96.79 ± 3.33 e	151.99 ± 3.78 c
	Total	467.97 ± 8.95 e	470.71 ± 9.15 e	424.36 ± 7.31 g	298.34 ± 3.71 i	449.02 ± 11.13 f	351.87 ± 6.48 h	895.48 ± 9.85 b	848.54 ± 19.49 c	787.29 ± 8.62 d	793.86 ± 6.04 d	1007.92 ± 16.10 a	863.51 ± 8.38 c

ND, not detected; Tr., trace amount detected but below quantification limit. Values represent the means ± SD from technical triplicate measurements. In each row, different letters mean significant differences (*p* ≤ 0.05).

## Data Availability

The data presented in this study are available on request from the corresponding authors.
